# Economic Behavior under the Influence of Alcohol: An Experiment on Time Preferences, Risk-Taking, and Altruism

**DOI:** 10.1371/journal.pone.0121530

**Published:** 2015-04-08

**Authors:** Luca Corazzini, Antonio Filippin, Paolo Vanin

**Affiliations:** 1 Department of Law Science and History of Institutions, University of Messina, Messina, Italy, and ISLA, Bocconi University, Milan, Italy; 2 Department of Economics, University of Milan, Milano, Italy, and Institute for the Study of Labor (IZA), Bonn, Germany; 3 Department of Economics, University of Bologna, Bologna, Italy; Institutes for Behavior Resources and Johns Hopkins University School of Medicine, UNITED STATES

## Abstract

We report results from an incentivized laboratory experiment undertaken with the purpose of providing controlled evidence on the causal effects of alcohol consumption on risk-taking, time preferences and altruism. Our design disentangles the pharmacological effects of alcohol intoxication from those mediated by expectations, as we compare the behavior of three groups of subjects: those who participated in an experiment with no reference to alcohol, those who were exposed to the possibility of consuming alcohol but were given a placebo and those who effectively consumed alcohol. All subjects participated in a series of economic tasks administered in the same sequence across treatments. After controlling for both the willingness to pay for an object and the potential misperception of probabilities as elicited in the experiment, we detect no effect of alcohol in depleting subjects’ risk tolerance. However, we find that alcohol intoxication increases impatience and makes subjects less altruistic.

## Introduction

There is a widespread consensus and social alarm about the potential costs of alcohol consumption, especially alcohol abuse. Alcohol is perceived to enhance risky behaviors and impulsive decision-making, thereby increasing the likelihood of unpleasant consequences for the consumer, as well as others with whom (s)he may come into contact. Traffic fatalities, gambling disorders, detrimental health conditions and risky sexual behaviors are only a few examples of the risks associated with alcohol abuse. According to the Fact Sheet 2011 of the World Health Organization “Alcohol is the world’s third largest risk factor for disease burden; it is the leading risk factor in the Western Pacific and the Americas and the second largest in Europe.”

The literature in both social sciences and medicine is rich in empirical studies that investigate the potential harmful behavioral consequences of alcohol consumption. Given its social relevance, a precise assessment of the causal behavioral effects of alcohol intoxication is important for the purposes of policy-making. The present paper contributes to this flourishing literature by reporting results from a laboratory experiment in which subjects participate in a battery of incentivized economic decision tasks in the domains of risk-taking, time preferences, optimism, value of money (willingness to pay for an object) and altruism (donations to social projects).

In addition to providing novel evidence regarding the pharmacological effects of alcohol intoxication on relevant economic domains, by using a laboratory technique, our study addresses important methodological issues. First, empirical studies of alcohol intoxication that are based on field data, whether collected from directly observed behavior or from self-reported answers, typically suffer from self-selection into the treatment, so they usually cannot identify the causal effects of alcohol consumption. The same is true for studies of drinking and binge drinking habits. Correlations between blood alcohol concentration and certain behavioral traits may reflect a true causal effect, but such correlations could also stem from variations in the propensity to drink alcohol by individuals with specific traits. Our study significantly limits the extent of the self-selection issue as participants to the experiment were recruited from the existing subject pool of the University of Milan; the only (weak) source of selection that remains in our design is due to the information in the inviting email about the possibility of consuming a moderate quantity of alcohol during the experiment. Moreover, by relying on a laboratory experiment that entails a random assignment to the treatment (alcohol), our analysis is able to identify the causal link between alcohol consumption and economic behavior. Second, individuals usually choose how much, when, where and with whom to drink alcoholic beverages at the same time, so it is usually difficult to disentangle the effects of alcohol from those of the context in which drinking takes place. Our results are instead free from the influence of the social environment, at the price of a modest loss of external validity because the subject pool is composed by students who volunteered to participate to the experiment. In this respect, there is a wide agreement in the profession that the external validity of laboratory experiment is not an issue when referred to the *qualitative* relationship between variables (see [1] and references therein). External validity becomes a serious concern for experiment conducted under a policy-evaluation perspective, i.e. aiming at providing a *quantitative* estimate of the relationship between variables, but this is not the goal of our paper. Third, the behavioral effects of alcohol intoxication are also partly pharmacological and partly triggered by a psychological reaction to the subjective perception of being under the influence of alcohol. Disentangling the two effects requires independent variations of actual and perceived blood alcohol concentration, in a similar way as [2] did in order to show the significant role played by expectations in mediating placebo effects of caffeine.

In this respect, a distinctive feature of our experiment is that it isolates the pharmacological effects of alcohol that are due to blood alcohol concentration from the psychological effects induced by the subjective misperception of intoxication. The pharmacological effect is identified by exploiting the variance between subjects who are treated with alcohol and those who receive a placebo beverage with no alcoholic content but whose perception is appropriately confounded. Our design also includes subjects who perform the same tasks as in the other two treatments but with no reference to alcohol.

Concerning risk preferences, after controlling for optimism, willingness to pay and other individual controls, we detect only a marginal positive effect of alcohol intoxication on risk aversion among female subjects. In contrast, we find a strong pharmacological effect of alcohol consumption on time preferences, as it makes subjects more impatient. The pure impact of alcohol consumption on time preferences remains large even after controlling for subjective measures of attitude toward risk, misperception of probabilities and willingness to pay. Finally, our results suggest that alcohol intoxication makes subjects more selfish, as it significantly reduces donations to humanitarian causes to which sober subjects contribute more substantial amounts.

### Alcohol, Risk and Impatience

Social scientists have devoted substantial effort in analyzing the relationship between alcohol consumption and risky behaviors in fields of investigation ranging from driving under the influence of alcohol and corresponding traffic fatalities [3, 4] to truancy and dropping our of high-school [5–7], from detrimental labor productivity and labor market outcomes of young adults [8] to health problems [9], and from risky sexual behavior [10, 11] to violent crimes [12]. Most of these studies are based on survey data and provide useful indications about the social costs associated with alcohol abuse.

A growing body of studies, based on the idea that (potentially) harmful behaviors are driven by risk preferences among other things, have investigated at the individual level the link between alcohol consumption habits and attitude toward risk. [13, 14] and [15], among others, show evidence of a significant correlation between risk aversion and alcohol consumption habits, but they also warn against causal interpretations of their findings.

A few studies investigate in the field individual-level correlation between alcohol intoxication and risk preferences, with mixed results. [16] find that alcohol makes women more risk-prone but that it has no effect on the risk propensity of male subjects. By contrast, [17] find that females’ risk aversion increases with both measured and perceived alcohol concentration, while only the latter has a positive correlation with males’ risk aversion. While field experiments improve the external validity of the results, they do not solve the problem of self-selection into drinking habits. Moreover, they make it difficult to disentangle the pharmacological impact of alcohol intoxication from context and peer effects [9, 18]. Peer effects have received increasing attention in the literature on alcohol abuse and risky behaviors, with studies focusing on the role played by fraternity membership [19, 20], social and family influence [21] and exposure to the “wrong” kinds of friendships [22].

Laboratory experiments offer a way to limit the self-selection issue and identify the causal effects of alcohol intoxication, but they have not yet provided conclusive results either. Some of these studies [23–26] report no relation or mixed evidence of alcohol on individual risk attitude, while others, such as [27], identify a positive pharmacological effect of acute alcohol intoxication on risk-taking. Laboratory experiments can also be used to disentangle the effects of alcohol from those of the drinking context. For instance, by making social interactions salient in their experimental design, [28] report that the positive effects of alcohol consumption on risk-taking behaviors are stronger when subjects act individually than when they participate in group decision-making.

There is also a growing literature that suggest that alcohol (and other substance) abuse tends to induce impulsive decision-making. Impulsivity is commonly defined in the psychological literature as the tendency to choose smaller and sooner rewards over larger and later ones, although larger and later rewards are preferred when the decision is not made in the “heat of the moment.” This form of preference reversal, which is usually explained in terms of hyperbolic discounting, has stimulated a large body of empirical research. Several studies have investigated whether substance abusers—and smokers in particular—are more impulsive than non abusers, finding largely supportive evidence [29, 30]. However, once again, correlation does not mean causality. On the one hand, subjects may become abuser because they are impatient, as [31] suggests. [32] support this interpretation by studying the acute alcohol consumption of subjects recruited in a pub. On the other hand, substance abuse may make subjects more impatient. [33] claims that alcohol addicted subjects are characterized by a steeper discounting of delayed rewards and [34] find similar evidence among adolescents. [35] document that college students who drink heavily discount hypothetical delayed rewards more steeply than light drinkers do. [36] find higher discount rates among heroin and cocaine users but not among alcoholics, as compared to controls. Again, controlled experiments are necessary to identify the direction of the causal link between time-discounting and alcohol consumption.

However, even randomized experiments have yielded no clear-cut results. [37] find that increased alcohol consumption leads to a counterintuitive increase in patience, whereas [38] find no significant effect of alcohol consumption on time preferences. In contrast, [39] find that subjects intoxicated at 0.8 g/kg performed more impulsively compared to the placebo in the Experiential Discounting Task (EDT), while no significant difference emerges between subjects intoxicated at 0.4 g/kg and the other two groups.

## Methods: Experimental Design

The experimental protocol was approved by the ethical committee of the University of Milan. The experiment involved voluntary students recruited using mailing list systems. The email used to recruit subjects for the sessions with reference to alcohol (see below) announced that the experiment could involve the consumption of a moderate quantity of alcohol. In this case, we explained that volunteers must have consumed alcohol before without experiencing any problems and that their physical and mental conditions did not advise against the consumption of a moderate amount of alcohol. Participants in the session with reference to alcohol were also reminded that they could withdraw from the study at any time. After having signed a consent form, a medical doctor assessed participants by means of anamnesis and a cursory physical examination to determine that they were suitable for the experiment.

Our experimental study investigates the causal effects of alcohol consumption on individual decision-making whereby we refer to individual decision-making as a situation in which strategic interaction plays no role because the consequences of one’s choices have no effect on other players. We compare results from two experiments: NO-ALC and ALC. In our benchmark, the NO-ALC experiment, subjects took incentivized economic decisions without consuming or hearing any reference to alcohol. The ALC experiment involved the same tasks as the benchmark, but before performing such tasks, subjects were required to drink a beverage, which they knew could contain alcohol. Some participants chosen at random received an alcoholic beverage while the remaining subjects received a non-alcoholic drink that smelled like alcohol. Thus, we distinguish among three treatments: the benchmark NO-ALC, whose subjects heard no reference to alcohol; the alcohol treatment ALC-T, whose subjects effectively consumed alcohol before taking economic decisions; and the *placebo* group ALC-P, whose subjects who did not consume alcohol but may have believed of being treated. We measured both actual and perceived blood alcohol concentration at various stages of the experiment.

We collected data from 3 ALC sessions and 3 NO-ALC sessions. 39 subjects participated in the ALC sessions and 38 subjects in the NO-ALC sessions, most of whom were undergraduate students of Economics. The experiment took place at the University of Milan between March and December 2011.

### Procedures

Upon their arrival at the experimental laboratory, subjects drew a number that was used to link them anonymously to final earnings. Once seated, subjects typed in their number into the computer, and at the end of the experiment, they were paid using envelopes marked with their numbers. The number was also used to assign subjects randomly to the two treatments, ALC-P and ALC-T. Subjects were never told to which treatment they were assigned. In fact, no reference was made at all to the two treatments; it was only publicly announced to all subjects in ALC that they would receive a beverage that might contain alcohol.

Before consuming the beverage, all participants were given a strong lozenge (“Fisherman’s Friend”) to make detection of the drink’s alcohol content (or lack thereof) more difficult. Subjects in ALC-T drank a mixture of peach juice and ethanol, with the amount of ethanol targeted according to the tables released by the Italian Health Ministry to reach average intoxication level of about 0.8 g/l, the legal limit for driving in several countries. Subjects in ALC-T received an average of 0.8 ml of ethanol per kg of body weight (measured at the beginning of the experiment), with the exact quantity varying between 0.67 ml/kg and 1 ml/kg, depending on gender, recent food intake and drinking habits. Those in ALC-P drank peach juice with 5 ml of grappa on the border of the glass and floated on the surface, a quantity that barely registers on a breath alcohol test but that gave the glass the characteristic smell of an alcoholic beverages. Our manipulation was designed to minimize the differences in the perceived alcohol intoxication between subjects in ALC-T and ALC-P in a deception-free environment. As documented by our descriptive statistics, it is difficult to grant equalization of subjects’ perception of alcohol intoxication between the two treatments. Nevertheless, the parametric analysis presented in the results accounts for this treatment heterogeneity and disentangles the pharmacological impact of alcohol intoxication from the effects that can be attributed to subjects’ mis-perception.

All participants were given 6 minutes to consume the beverage and were instructed to stop drinking right away if they experienced any unpleasant effect. In the 30 minutes after drinking—that is before alcohol consumption could alter their understanding of the tasks—one researcher read aloud the instructions of the elicitation mechanism proposed by [40] (BDM hereafter; see the next section for details).

Immediately before starting their tasks, participants’ Blood Alcohol Concentration (BAC) was measured with a Lion500 professional alcoholmeter and results were never announced. Subjects were also asked to self-report what they believed their intoxication level to be using the same alcoholmeter scale. Overall, this process lasted about 15 minutes. Measured and perceived BAC recorded in the *MBAC* and *PBAC* variables, respectively, allow us to identify both the pharmacological and the indirect, expectation-mediated effects of alcohol consumption on subjects’ economic decisions.

The NO-ALC experiment was identical to the ALC experiment, but for the removal of any reference to alcohol. Subjects in the NO-ALC experiment did not consume any alcohol, and alcohol was never mentioned either in the recruiting email or during the experiment. To remove any reference to possible intoxication, subjects in the NO-ALC experiment did not meet the doctor for the anamnesis and the check, nor had their measured and perceived BAC recorded. To minimize the difference in time between the two experiments, we implemented the same BDM briefing stage in NO-ALC as in ALC. The two experimental designs coincided in all other respects.

### The Tasks

After the introductory phase in both ALC and NO-ALC, subjects took consecutive, non-strategic economic decisions in the domains of attitude toward risk, willingness to pay for an object, altruism, optimism and impatience. We retained the same order of economic decisions in all experiments so any differences in behaviors between ALC and NO-ALC could be imputed only to the alcoholic treatment. The tasks were computerized using z-Tree [41], a flexible computer platform that is widely used in experimental economics to administer incentivized tasks in networks. The choice of the tasks was made to cover a large spectrum of aspects of individual decision-making studied in the economic literature. The tasks were implemented in an incentive-compatible way. Subjects were told that, although they were going to make several economic decisions, their final payments would depend on a single task randomly selected at the end of the experiment. In calibrating the parameters involved in the tasks, we made an effort to ensure that the potential earnings were comparable across tasks.

Phases 1, 2 and 6 were based on the BDM elicitation mechanism, an incentive-compatible device used to elicit reservation prices. Under BDM, an individual reports a bid (ask) for an item. Then, the item’s price is randomly drawn. If the bid (ask) is above (below) the price, the subject receives (sells) the good and pays (receives) the drawn price. If the bid (ask) is below (above) the price, the subject does not receive (sell) the good and pays (receives) nothing. The detailed explanation of the BDM mechanism that was provided to the subjects and the English instructions for the experimental tasks are included in the Supporting Information (see S1 Experimental Instructions). An important feature of BDM that makes this device useful for our purposes is that it can be adapted easily to a variety of economic tasks.

#### Risk attitude (Phase 1)

We measured the subjects’ risk attitude by eliciting their ask prices in a battery of 10 lotteries through the BDM mechanism. Lotteries entail the same events (0-euro gain vs. 40-euro gain) with various probabilities of winning, ranging from 10% to 100%. Lotteries were presented in the same randomly prearranged order to reduce anchoring effects that might induce risk neutrality. This elicitation method is not the most widely used, and it has been criticized because of the high cognitive load required [42]. Although complexity is definitely one of the most relevant dimensions along which the elicitation tasks should be evaluated [43], the design of our experiment exposes subjects repeatedly to the BDM mechanism, so the use of BDM to measure risk attitude implies no additional cognitive load at the margin, which would have happened had we chosen another risk-elicitation method that required additional instructions (such as the task introduced by [44]). Moreover, as already mentioned, subjects were appropriately briefed in the introductory phase on how BDM works, reducing the cognitive load. This task also has the advantage of allowing us to measure subjects’ risk attitude along the whole domain of probabilities. As a summary measure of risk attitudes, we used the average difference between the expected value and the ask price (or selling price) across all the lotteries. According to standard rational choice models of individual decision-making, this variable—called *Risk aversion* hereafter—should be equal to zero, positive and negative for risk-neutral, risk-averse and risk-loving individuals, respectively. One of our main research questions concerns the effect of actual or perceived BAC on *Risk aversion*.

#### Willingness to pay (Phase 2)

In this phase, subjects were endowed with 20 euro. We measured their willingness to pay for a radio-videogame using the BDM and recorded their bid price. This variable—*WTP* hereafter—reflects the value (or marginal utility) that is subjectively attributed to money, and should be distributed in the same way across random samples of the population. The design of monetary incentives in experiments is usually based on the assumption of constant marginal utility of money in order to keep incentives constant and identify the causal effects of a treatment. In the context of our experiment, this assumption cannot be taken for granted, as actual or perceived alcohol exposure could alter subjects’ willingness to spend money, possibly threatening our ability to identify the causal effects of alcohol on our variables of interest: altruism, risk and time preferences. It is thus important to control for differences in *WTP* across treatments.

#### Altruism (Phases 3 and 4)

In each of these two phases, subjects were endowed with 20 euro and were involved in a dictator game, in which they chose how much to donate (if anything) to a non-governmental organization (NGO). The two phases differed in the selected NGO: the humanitarian aid agency Mèdecins Sans Frontiéres (MSF) in one case and the non-humanitarian Italian independent website of economic information LaVoce.info (LV) in the other. We measured altruism as the amount donated to these two NGOs, respectively recorded in the *Altruism MSF* and *Altruism LV* variables. We investigate whether and how alcohol affects altruism, particularly whether its effect on the propensity to make donations to the humanitarian (MSF) and the non humanitarian (LV) causes differs. This analysis complements the study of the effects of alcohol on risk and time preferences and extends it to social preferences.

#### Optimism (Phase 5)

We assessed subjects’ optimism by randomly extracting a sample of 21 covered cards out of a maze of 52 poker cards. Each participant drew one card from the extracted sample and were told that they would win 1 euro for each card in the sample that had the same color as his or her card. Subjects then reported how many of the 21 cards they expected to be the same color as their own. Such beliefs, captured by a variable called *Optimism*, were elicited in an incentive compatible way by assigning a prize of 10 euro if they turned out to be correct. Due to the random selection draw, the expected value is that the drawn sample contains 10.5 cards of any color, so higher levels of *Optimism* reflect an optimistic view of one’s chances of earning money. This task offers an important control for the study of the effects of alcohol on risk attitude, as, if alcohol exposure alters the perception of probabilities, the actual or perceived alcohol intoxication could modify subjects’ propensity to make risky choices—not because of a change in risk preferences, but because of a change in optimism about the likely consequences. The variable *Optimism* allows us to control for this possibility.

#### Impatience (Phase 6)

In this phase, each subject was given a cash card in which the experimenters would transfer 20 euro one hour after the end of the experiment. We measured participants’ impatience by asking them how much money they would require in order to postpone the money transfer by one, seven, and eight days. Such requests, recorded as *One day*, *Seven days* and *Eight days*, respectively, were made incentive-compatible through the BDM mechanism. The use of a cash card allowed us to measure subjects time preferences by cleanly controlling for trust and transfer costs. We chose to implement the impatience task at the end of the experiment to enhance the salience of postponing the receipt of money by minimizing the time between the task and the final payments.

### Debriefing

**Fig 1:**
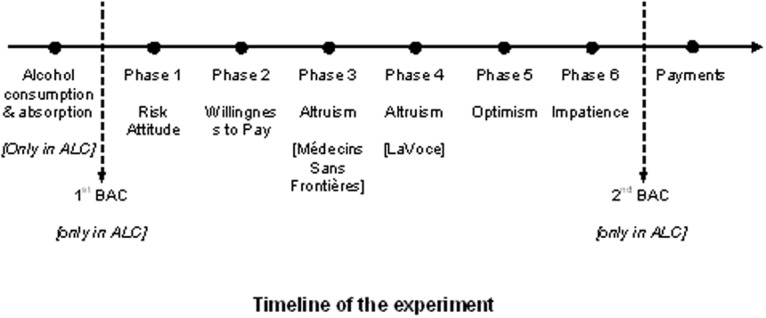
Timeline of the Experiment.

The data-collection phase lasted about 30 minutes and finished with subjects filling in an anonymous questionnaire to gather demographic and socio-economic information and some self-reported measures of happiness and trust. Following data collection, participants in ALC had their measured and perceived BAC recorded for the second time (*MBAC2* and *PBAC2*), with subjects still unaware of the result of the first measure (*MBAC* and *PBAC*). Individual payoffs were then determined according to the specific rules of the phase randomly drawn for payment in each session. Participants in ALC who had a measured BAC above 0.5 g/l were invited to remain in the laboratory until their BAC decreased below the legal limit allowed to drive in Italy, and we suspended the payment until that time. Before they left the laboratory, all subjects were asked to sign a statement that declared that they felt physically and mentally comfortable and that they perceived no impairment following their participation in the experiment. Fig. 1 summarizes the timing of the experiment.

## Results

On average, subjects earned 13.85 euros in sessions that lasted about 90 minutes in the case of ALC and 70 minutes in the case of NO-ALC sessions. Table 1 presents the summary statistics for age, gender, and performance in the various tasks for ALC-T, ALC-P, and NO-ALC, separately.

**Table 1:** Summary Statistics.

Treatment	ALC-T (20 Obs)				ALC-P (19 Obs)				NO-ALC (38 Obs)			
Variable	Mean	St.Dev	Min	Max	Mean	St.Dev	Min	Max	Mean	St.Dev	Min	Max
*Age*	23.2	2.191	20	30	23.053	1.649	21	27	24.605	5.38	19	45
*Female*	.4	.503	0	1	.263	.452	0	1	.579	.5	0	1
*WTP*	3.145	3.852	0	12	3.916	4.531	0	18.8	7.029	4.941	0	20
*Optimism*	10.55	1.701	7	13	10.789	2.371	6	13	11.711	2.779	8	21
*Risk aversion*	1.636	7.078	-8.5	19.1	.592	4.218	-9.3	8.5	1.536	5.251	-4.2	17.6
*One day*	7.475	7.236	.5	20	4.342	3.87	0	13	5.403	5.592	0	20
*Seven days*	11.125	6.011	2.5	20	7.839	4.511	0	17	8.388	5.837	0	20
*Eight days*	13.095	6.272	3	20	9.742	5.547	0	20	9.618	6.582	0	20
*Donation MSF*	4.645	5.009	0	15	7.184	6.176	0	20	9.5	5.831	0	20
*Donation LV*	2.8	4.053	0	15	2.395	2.313	0	6	4.982	4.022	0	15

### Measured and Perceived BAC

The differences in BAC induced by the random allocation of subjects in ALC to the alcohol (ALC-T) and placebo (ALC-P) treatments are shown in Table 2, which presents—by treatment—the summary statistics of measured and perceived BAC at the first (*MBAC* and *PBAC*) and second (*MBAC2* and *PBAC2*) recording. The table also displays summary statistics for misperceived BAC, defined as the difference between perceived and measured BAC, at the two recording times (i.e., *MPBAC = PBAC − MBAC* and *MPBAC2 = PBAC2 − MBAC2*). This variable captures the belief that one is intoxicated that is not backed by actual BAC. For instance, a value of *MPBAC* > 0 indicates that the subject overestimates her or his intoxication level. Misperceived BAC is used as a measure for the placebo effect of alcohol.

**Table 2:** Measured and Perceived BAC by Treatment.

Treatment	ALC-T (20 Obs)				ALC-P (19 Obs)			
Variable	Mean	Std. Dev.	Min	Max	Mean	Std. Dev.	Min	Max
*MBAC*	.596	.159	.32	.91	.017	.03	0	.08
*MBAC2*	.557	.114	.32	.77	.01	.024	0	.07
*PBAC*	.715	.319	.2	1.5	.369	.246	0	.8
*PBAC2*	.493	.218	.09	1	.241	.214	0	.65
*MPBAC*	.119	.32	-.38	.75	.352	.234	0	.74
*MPBAC2*	-.064	.235	-.47	.63	.231	.209	0	.6

*Notes:* These variables are equal to zero by construction in the NO-ALC treatment.

As no specific reference to alcohol is made in the NO-ALC experiment, we set both *MBAC* and *MPBAC* measures equal to zero for these participants. We use *MBAC* and *MPBAC*, along with a dummy for the NO-ALC experiment, called *NO-ALC*, as our main explanatory variables. This approach is preferable to the alternative of using *MBAC* and *PBAC* (always with the *NO-ALC* dummy), because, although statistically equivalent, it allows disentangling the pharmacological and the placebo effect of alcohol in a cleaner way. At the same time, it also allows recovering the significance of the effect of perceived intoxication through simple econometric tests.

**Fig 2:**
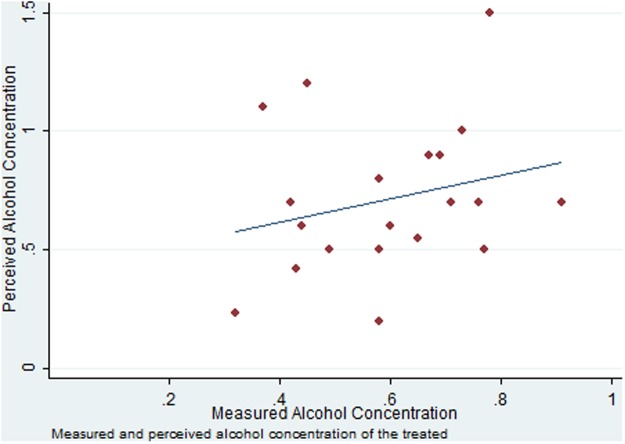
Measured and Perceived Alcohol Concentration of the treated.

At both recording times, perceived and measured BAC are significantly higher in ALC-T than in ALC-P. A Mann-Whitney test rejects the null hypothesis that both *MBAC* and *PBAC* are equal in the two treatments (*p* < 0.0001 and *p* = 0.0009, respectively). An analogous test for *MBAC2* and *PBAC2* rejects equality between the two treatments with *p* < 0.0001 and *p* = 0.0018, respectively. At the first recording, before performing the incentivized tasks, subjects in both treatments tend to overestimate their intoxication level, as the average *PBAC* is significantly higher than *MBAC*. At the second recording, after performing the tasks, subjects in both treatments revise their perceived BAC downwards. In ALC-T, the revision in perception substantially overestimates the objective decrease in measured BAC, with *PBAC2* becoming lower than *MBAC2* on average. In ALC-P, the perceived BAC remains high even though subjects did not actually consume alcohol. Fig. 2 shows the distribution of the first recording of measured and perceived BAC for the ALC-T subjects.

**Fig 3:**
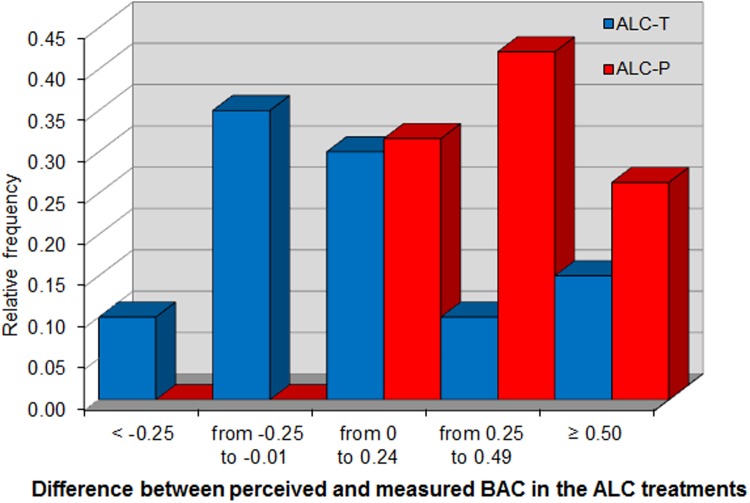
Misperception of Blood Alcohol Concentration (MPBAC).

At both recording times, *MPBAC* is significantly higher in ALC-P than in ALC-T. A Mann-Whitney test rejects the null hypothesis that *MPBAC* is equal in the two treatments with *p* = 0.0064 (for *MPBAC2* equality is rejected with *p* < 0.0001). Fig. 3 shows the distribution of *MPBAC* in the two ALC conditions. Our design has been effective in inducing among the participants in ALC-P the belief that they had consumed alcohol. This offers a good basis on which to identify the pharmacological and placebo effects of alcohol, since the design reduces the strongly positive correlation between perceived and actual alcohol intake that would have been observed otherwise.

Since perceived BAC at the second recording may be endogenous to performance in the experiment, we base our analysis on the first recording. We have also run all the regressions with BAC measures from the second recording, obtaining results that are highly aligned because of the high correlation between the two recording times (0.978 for *MBAC* and *MBAC2*, 0.918 for *PBAC* and *PBAC2*; these results are available upon request but are not included here because the endogeneity issue renders them less interpretable).

We have also run all the regressions using *ALC-T* (a dummy for the alcohol treatment) and *NO-ALC* as the main explanatory variables (in place of *MBAC*, *MPBAC* and *NO-ALC*). We refer to this analysis as to treatment dummy regressions. While disregarding information on the intensive margin of alcohol intoxication results in a slight loss of explanatory power and precision, it allows a clearcut comparison of the different treatments. Results, which confirm those obtained exploiting the intensive margin of intoxication, are not reported in detail for the sake of space, although we refer to them whenever useful. Since the excluded dummy is the one for the placebo treatment, *ALC-P*, the coefficient of *ALC-T* measures the differential impact (on each dependent variable) of the alcohol treatment relative to the placebo, whereas the coefficient of *NO-ALC* captures the differential impact between the placebo and no-alcohol treatment, that is, they capture the pharmacological and placebo effects of alcohol, respectively. These results are included in the Supporting Information (see S1 Additional Regressions).

### Alcohol, Monetary Incentives and Optimism

Before looking at the effects of alcohol on risk-taking, time preferences and altruism, it is worth considering its impact on *WTP* and *Optimism*, which will be used as controls in the subsequent analysis.

The comparatively large monetary incentives involved in the experiment contribute to the methodological validity of our design. Nevertheless, the assumption that subjects who differ in terms of their alcohol consumption perceive monetary incentives in the same way cannot be taken for granted. To determine whether subjects with higher BAC (measured or perceived) value money differently from subjects with lower or no alcohol exposure, we compare the subjects willingness to pay for a given object (a radio-videogame) in Phase 2 of the experiment.

Columns (1) and (2) of Table 3 show the ordinary least squares (OLS) estimates from a linear model in which the willingness to pay, *WTP*, is regressed against *MBAC*, *MPBAC* and *NO-ALC*, first without and then with additional exogenous individual controls.

**Table 3:** Alcohol, Value of Money and Optimism.

Dependent variable	*WTP*	*WTP*	*Optimism*	*Optimism*
	(1)	(2)	(3)	(4)
*MBAC*	-.3290	-.4036	-.7282	-.6894
	(1.0257)	(.8093)	(.6841)	(.8120)
*MPBAC*	.0307	.6089	-1.9665**	-1.5074
	(1.0340)	(.9757)	(.7989)	(.9584)
*NO-ALC*	3.4119**	3.1607*	.3581	.4168
	(1.5996)	(1.6485)	(.5163)	(.6322)
*Age*		.0499		-.0878***
		(.0874)		(.0215)
*Female*		1.1739*		.7731
		(.6944)		(.5509)
*Constant*	3.6168***	1.9608	11.3524***	13.0064***
	(1.2890)	(2.6524)	(.4891)	(.8498)
Observations	77	77	77	77
R-Square	.132	.1477	.0691	.1065

*Notes:* OLS regressions. The dependent variable in columns (1) and (2), *WTP*, is the willingness to pay for the object in Phase 2 of the experiment; the dependent variable in columns (3) and (4), *Optimism*, is the expected gain in the card game (elicited in Phase 5). *MBAC* is the measured level of Blood Alcohol Concentration (BAC), *MPBAC* is the difference between perceived and measured BAC, *NO-ALC* is a dummy for the NO-ALC experiment. *Age* is respondents’ age (in years) and *Female* is a gender dummy. Significance level (***: 1%; **: 5%; *: 10%) based on robust standard errors (reported in parenthesis), clustered at the experimental session level (8 clusters).

It emerges that subjects are significantly more willing to pay for the objects when no reference is made to alcohol. A possible interpretation of this result is that, in the NO-ALC condition, subjects do not bear the risk of consuming alcohol, so they may consider the experiment as requiring less effort than their counterparts in the ALC treatments might. Consequently they could dispose more easily of their money because they perceive it as windfall gains. This interpretation is also consistent with the findings about altruism reported below. An alternative explanation is that exposure to alcohol triggers self-control mechanisms on expenditures. In turn, when participants are exposed to alcohol consumption (ALC), differences in *MBAC* and in *MPBAC* do not translate into significant differences in how the participants value money. These results are confirmed by non-parametric analysis. A Mann-Whitney test rejects the null hypothesis that the average *WTP* is the same for NO-ALC and ALC (*p* = 0.0007) and for NO-ALC and ALC-P (*p* = 0.0101), whereas equality between ALC-T and ALC-P cannot be rejected (*p* = 0.4561). Once controlling for age and gender, treatment dummy regressions show no significant effect of alcohol on *WTP*, whether pharmacological or placebo. While this result is already reassuring for the validity of the comparison between subjects in treatments ALC-T and ALC-P, we always control for individual *WTP* in the regression analysis below, finding that it is never significant and its inclusion never alters the sign, the magnitude, or the significance of the effects of alcohol.

It is also worth noting that the variance of *WTP* is lower in ALC-T than in the other treatments (see Table 1). This suggests that the levels of alcohol intoxication observed in the experiment neither introduce additional noise to subjects’ choices nor impair their ability to make rational choices. Again, this results reassuring for the possibility of interpreting subsequent results within the rational choice framework.

The assessment of the effects of alcohol on risk aversion also relies on the assumption that alcohol does not alter the perception of probabilities (in particular, perception of the odds of winning). We investigate the validity of this assumption by observing the effects of alcohol intoxication on subjects optimism, as elicited in Phase 5. If alcohol affects optimism, the analysis of risk aversion should control for this effect, just as it is important to control for the value of money.

Columns (3) and (4) of Table 3 show OLS estimates of *Optimism* regressed against the same set of controls used in the first two columns. While there is some evidence of a placebo effect, with subjects who overestimate their BAC expressing more pessimistic guesses, such evidence is not robust to the introduction of controls. Non-parametric analysis confirms the absence of any difference in *Optimism* across treatments. A Mann-Whitney test cannot reject the null hypothesis that *Optimism* is the same for ALC-T and ALC-P (*p* = 0.3615), for NO-ALC and ALC-P (*p* = 0.5138), and for NO-ALC and ALC-T (*p* = 0.1606). Once controlling for age and gender, treatment dummy regressions show no significant effect of alcohol on *Optimism*, whether pharmacological or placebo.

### Alcohol and Risk Preferences

We use the selling (ask) price of the lotteries elicited through the BDM procedure in Phase 1 to measure subjects attitude toward risk. A risk-neutral agent should evaluate any lottery exactly at its expected value, while risk-averse (risk-loving) agents should instead ask lower (higher) prices. For each individual, we use as a summary measure of *Risk aversion* the average difference, across the ten lotteries, between the expected value and the selling price. On average, experimental subjects have a slight degree of risk aversion, with the average expected value and selling price of 22 euro and 20.66 euro, respectively. The average amount that subjects are willing to forgo to avoid the risk associated with lotteries is 1.34 euro, that is, 6% of expected gains. As shown in Table 1, there are only minor differences across treatments: the average *Risk aversion* is 1.64, 0.59, and 1.54, respectively, that is 7.4%, 2.7%, and 7% of expected gains. Therefore, unlike what has been observed in several other lab experiments, our subjects have preferences that are, on average, close to risk neutrality, which is in line with Rabin’s calibration theorem [45].

**Fig 4:**
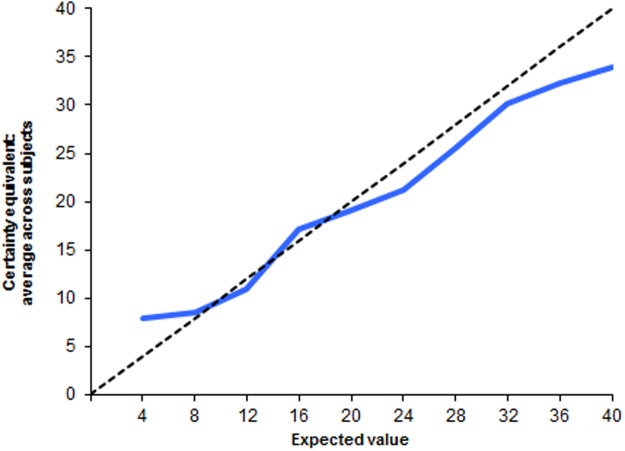
Average certainty equivalent (selling price) by lottery.

The selling price tends to be lower than the expected value particularly in the lotteries with high probabilities of winning, as shown in Fig. 4. The pattern around the expected value of the lotteries is consistent with the estimated shape of the probability-weighting function [46].

Our key research question concerns whether risk attitude is affected by alcohol intoxication and whether it changes across the experimental treatments. Columns (1) and (2) of Table 4 show the OLS estimates of *Risk aversion* regressed against the same set of controls used in Table 3—that is, the treatment variables *MBAC*, *MPBAC* and *NO-ALC*—first without the exogenous individual controls *Age* and *Female*, and then with them. Common wisdom might suggest that alcohol increases risk-seeking behavior, but our multivariate framework contradicts this conjecture, as no treatment variable is significantly related to *Risk aversion*. The same is true for perceived BAC, which is simply *PBAC* = *MBAC*+*MPBAC*: a Wald test cannot reject the null hypothesis that *MBAC*+*MPBAC* = 0 (*p* > 0.5 in all specifications). Females appear to have a significantly higher degree of risk aversion than males do. Column (3) adds individual controls for the marginal utility of money and for optimism: the coefficients of *WTP* and *Optimism* are not significantly different from zero and their inclusion does not alter the previous results. Treatment dummy regressions confirm that there is no significant effect of alcohol on *Risk aversion*, whether pharmacological or placebo.

**Table 4:** Alcohol and Risk Preferences.

Dependent variable	*Risk aversion*	*Risk aversion*	*Risk aversion*
	(1)	(2)	(3)
*MBAC*	-.2790	-.2739	.0316
	(1.6759)	(1.9600)	(2.2376)
*MPBAC*	-2.7993	-1.0813	-.7754
	(3.1056)	(2.9742)	(2.8707)
*NO-ALC*	-.3298	-.4787	-1.3713
	(1.7146)	(1.9179)	(1.4418)
*Age*		-.1467	-.1324
		(.1295)	(.0896)
*Female*		3.1197***	2.6021**
		(.8948)	(1.1566)
*WTP*			.2427
			(.1502)
*Optimism*			.3010
			(.4116)
*Constant*	1.8654*	3.8177	-.5727
	(1.0368)	(3.3130)	(3.5742)
Observations	77	77	77
R-Square	.0122	.0888	.1452

*Notes:* OLS regressions. The dependent variable, *Risk aversion*, is the average difference between the expected value and the ask price across the ten lotteries elicited in Phase 1 of the experiment. Regressors are defined in the notes to Table 3. Significance level (***: 1%; **: 5%; *: 10%) based on robust standard errors (reported in parenthesis), clustered at the experimental session level (8 clusters).

Table 5 splits the average *Risk aversion* by gender and treatment, showing how the gender effect on risk attitudes is mediated by alcohol intoxication. In the NO-ALC and ALC-P treatments—that involved no alcohol consumption—the choices made by males and females are similar on average and not far from those associated with risk neutrality. By contrast, in ALC-T, where participants do consume alcohol, the behavior diverges sharply: while males have slightly risk-seeking preferences, females’ risk aversion increases dramatically, as they are willing to give up almost 30% of the expected gain to avoid uncertainty.

**Table 5:** *Risk aversion* by Treatment and Gender.

Treatment	Males	Females
NO-ALC	.77	2.10
ALC-P	.29	1.44
ALC-T	-1.49	6.32

Splitting the sample along two dimensions (treatment and gender) results in a further reduction in the number of observations from which to make inferences. For this reason, most of the comparisons by treatment and gender are not significant, with the remarkable exception of the gender differences in risk aversion in the ALC-T condition (*p* = 0.0370). A similar argument applies when interacting gender with measured and misperceived BAC (*MBAC* and *MPBAC*) in a multivariate framework. The level of measured alcohol intoxication is positively associated with risk aversion, with this effect being marginally significant for females and not significant for males; for the sake of space, this result is not reported, but it is available upon request. Therefore, what emerges is a gender-specific compensation effect, with only intoxicated female subjects compensating for the detrimental effects of alcohol by displaying more prudent behavior. This finding is in line with [47], who also detect a compensation effect for females in a cognitive task. Similar risk attitudes emerge for subjects who are not exposed to alcohol consumption (NO-ALC and ALC-P), which is consistent with the recent findings of [48], who argue that gender differences are usually not observed with elicitation methods that are characterized by changing probabilities and the absence of a riskless alternative like the BDM.

### Alcohol and Time Preferences

**Fig 5:**
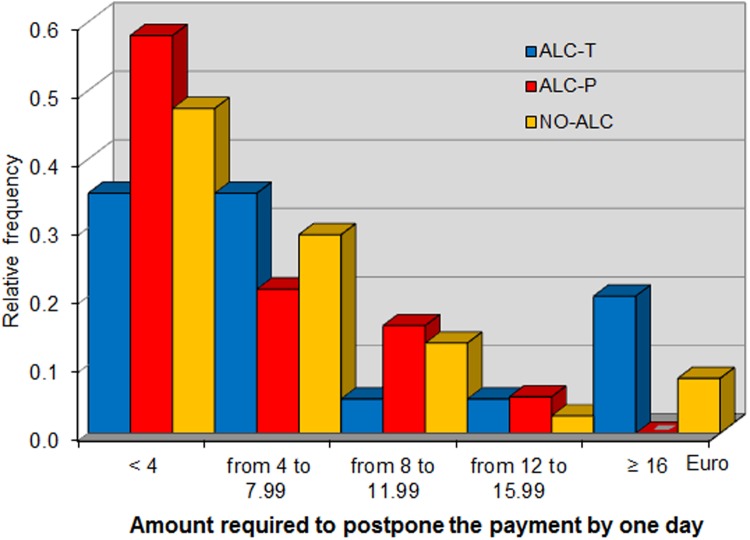
Distribution of additional sum required to postpone payment by one day.

**Fig 6:**
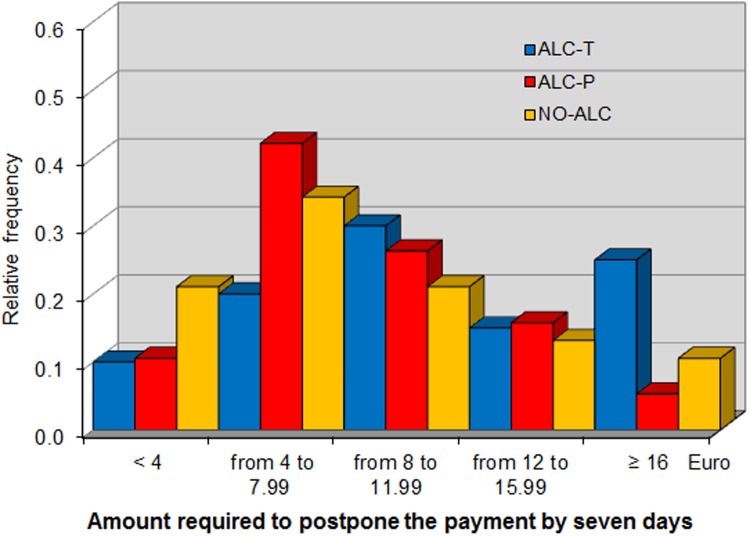
Distribution of additional sum required to postpone payment by seven days.

*One day*, *Seven days* and *Eight days* refer to the additional sums required to postpone payment by the respective number of days (elicited in Phase 6 of the experiment). Fig. 5, 6, and 7 show the distribution of these variables across the different treatments, showing that alcohol consumption makes individuals more impatient. In fact, the distribution of premiums requested by subjects in the in ALC-T treatment at seven and eight days stochastically dominates that of those in the ALC-P and NO-ALC treatments. This result is confirmed by non-parametric analysis. At any future date, the average delay premium is higher in ALC-T than in ALC-P, with their difference being significant at seven and eight days. In particular, a battery of Mann-Whitney tests marginally rejects the null hypothesis that the average of *Seven days* is the same for ALC-T and ALC-P (*p* = 0.0984), and the same is true for *Eight days* (*p* = 0.0985). By contrast, the same tests cannot reject equality of these variables between NO-ALC and ALC-P (*p* = 0.9323 and *p* = 0.8653, respectively), nor can they reject equality of *One day* between ALC-T and ALC-P (*p* = 0.1846).

**Fig 7:**
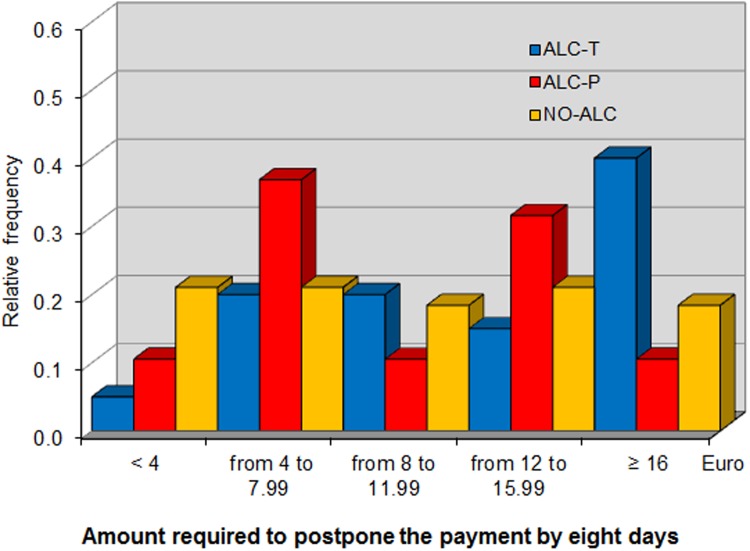
Distribution of additional sum required to postpone payment by eight days.

Our data can be used to calculate individual discount factors (relative to the present) at different points in time. Assuming that individual utility is linear in income and additively separable in time, the present discounted utility of a stream of income **y** = (*y*_0_, …, *y*_*T*_), from time 0 to *T*, can be expressed as $U(\text{y})=\sum_{t=0}^{T}\beta_{t}y_{t}$. The assumption of constant marginal instantaneous utility of money is common in the literature, and it is a plausible approximation in light of the small degree of risk aversion we find in the data. In any case, to account for possible deviations from risk neutrality, we include individual risk aversion among the controls in later regression analysis. Multiplying discounted utility by a constant to rescale from monetary to utility units is irrelevant for choice, so it is omitted. Standard neoclassical economics assumes exponential discounting: *β*_*t*_ = *β* ^*t*^, for some *β* ∈ (0, 1). This functional specification implies that the ratio between the discount factors of two adjacent moments in time is always constant: for any *t*, *β*_*t*+1_/*β*_*t*_ = *β*. Rational individuals who discount the future exponentially should make time-consistent choices. Behavioral economists have often explained the abundant evidence of time-inconsistent choices based on the idea that the discount factor is a hyperbolic or quasi-hyperbolic (rather than exponential) function of time, which means that *β*_*t*_ drops faster from one period to the next when the present is near (*t* is close to zero) than when it is not (*t* is high). Our experiment allows exponential versus hyperbolic discounting to be tested. Since we have data for *t* ∈ {0, 1, 7, 8}, and since *β*_0_ = 1 by construction such that *β*_1_/*β*_0_ = *β*_1_, we test the null hypothesis that *β*_1_ = *β*_8_/*β*_7_ against the alternative that *β*_1_ < *β*_8_/*β*_7_. We find that *β*_1_ = 0.8108 and *β*_8_/*β*_7_ = 0.9589. The null hypothesis that they are equal is rejected by a t-test at the 1% significance level in both the full sample and the subsamples that correspond to each treatment, suggesting that our subjects discount the future in a hyperbolic-like way, rather than an exponential way (or a current discussion of the literature on hyperbolic discounting and for an alternative interpretation in terms of subjective time compression, see [49]).

To continue in our investigation of the effects of alcohol on time preferences, we construct a panel using as the longitudinal dimension the individual discount factor at several points in time (relative to the present). Thus, we define the variable *Patience*, which includes four observations for each individual, calculated as follows: *β*_0_ is set equal to one by construction, whereas for *t* ∈ {1, 7, 8}, *β*_*t*_ = 20/*y*_*t*_, where *y*_*t*_ is the overall amount received at time *t* (20 euros plus the amount required to postpone the payment at each future date), as elicited in Phase 6 of the experiment.

The experimental design is structured so that rational and risk-neutral agents ask for overall future sums *y*_*t*_, whose present discounted value is equal to the current payment, which is 20 euros: *β*_*t*_ *y*_*t*_ = 20. The panel structure of our dataset allows us to run a random effect estimation of the longitudinal variable *Patience* against the main regressors (*MBAC*, *MPBAC* and *NO-ALC*, which are longitudinally constant for each individual) and a number of additional controls. Results are shown in Table 6 in which standard errors are clustered at the subject level in order to take into account the dependence of the three choices made at 1, 7, and 8 days.

**Table 6:** Alcohol and Time Discounting, Panel.

Dependent variable	*Patience*	*Patience*	*Patience*	*Patience*	*Patience*	*Patience*
	(1)	(2)	(3)	(4)	(5)	(6)
*MBAC*	-.1084**	-.1084**	-.1066**	-.1034**		
	(.0476)	(.0478)	(.0478)	(.0442)		
*MPBAC*	-.0842	-.0842	-.0807	-.0709	-.0615	
	(.0519)	(.0521)	(.0550)	(.0582)	(.0544)	
*NO-ALC*	-.0296	-.0296	-.0254	-.0235	-.0148	-.0138
	(.0317)	(.0318)	(.0319)	(.0310)	(.0227)	(.0195)
*1D*		-.1892***	-.1892***	-.1892***	-.1759***	-.1698***
		(.0170)	(.0171)	(.0171)	(.0185)	(.0213)
*7D*		-.2835***	-.2835***	-.2835***	-.2659***	-.2572***
		(.0158)	(.0159)	(.0160)	(.0184)	(.0204)
*8D*		-.3150***	-.3150***	-.3150***	-.2935***	-.2816***
		(.0174)	(.0174)	(.0175)	(.0206)	(.0225)
*Age*			-.0025	-.0015	-.0015	-.0015
			(.0039)	(.0037)	(.0037)	(.0037)
*Female*			.0035	-.0155	-.0157	-.0152
			(.0232)	(.0221)	(.0222)	(.0215)
*WTP*				-.00008	-.00006	-.00007
				(.0019)	(.0019)	(.0019)
*Risk aversion*				.0055***	.0055***	.0055***
				(.0019)	(.0019)	(.0019)
*Optimism*				.0025	.0026	.0027
				(.0057)	(.0057)	(.0056)
MBAC*1D					-.0834	-.0832
					(.0637)	(.0632)
MBAC*7D					-.1104**	-.1107**
					(.0501)	(.0490)
MBAC*8D					-.1350***	-.1359***
					(.0506)	(.0486)
MPBAC*1D						-.0522
						(.0730)
MPBAC*7D						-.0734
						(.0608)
MPBAC*8D						-.1001
						(.0668)
*Constant*	.8448***	1.0418***	1.0978***	1.0440***	1.0216***	1.0133***
	(.0269)	(.0258)	(.0915)	(.1157)	(.1138)	(.1112)
Observations	308	308	308	308	308	308
Overall R-Square	.022	.5121	.5154	.5448	.5495	.5519

*Notes:* Random effect panel estimate. The dependent variable, *Patience*, is the discount factor at different dates (set equal to one for the present and measured in one, seven and eight days by the ratio of the present payment –20 euros– to the total amount required to postpone payment at each future date, as elicited in Phase 6 of the experiment), constructed as a longitudinal variable with four observations for each individual. *1D*, *7D* and *8D* are dummies for the respective future dates. MBAC*1D, MBAC*7D and MBAC*8D, and MPBAC*1D, MPBAC*7D and MPBAC*8D, are interaction terms between these dummies and *MBAC* and *MPBAC*, respectively. The other regressors are defined in the notes to Table 3 and Table 4. Significance level (***: 1%; **: 5%; *: 10%) based on robust standard errors (reported in parenthesis), clustered at the individual level (77 clusters).

Column (1), which considers only the main determinants of interest, confirms that alcohol consumption raises impatience, i.e it reduces *Patience*, both through a (significant) pharmacological effect and through a (non significant) placebo effect. A Wald test rejects the null hypothesis *MBAC*+*MPBAC* = 0 at the 5% significance level. This result, which remains true throughout the different specifications from column (1) to (4), means that perceived alcohol intoxication is negatively and significantly associated to *Patience*. Table 6 disentangles the effect of perception (*PBAC*) into that of its two virtually orthogonal components, namely measured alcohol intoxication (*MBAC*) and its misperception (*MPBAC*), and shows that only the former significantly explains variations in impatience.

Column (2) exploits the longitudinal dimension and introduces three time dummies for future dates, *1D*, *7D* and *8D*, corresponding to one, seven and eight days from the date of the experiment, respectively. While introducing these dummies does not affect the results on the effects of alcohol, it confirms the result on the hyperbolic shape of time-discounting. Contrary to the constant discount rate predicted by exponential discounting, postponing the payment by one day makes the discount factor drop faster when it takes place at present than it does if it takes place in seven days. As shown by Column (3), results are essentially unaffected by the introduction of additional (exogenous) individual controls.

Column (4) introduces our experimental measures of willingness to pay, risk aversion, and optimism, showing that *Patience* is significantly and positively correlated with *Risk aversion*, but it is not significantly associated with the other two variables. The positive sign of *Risk aversion*’s coefficient supports the claim that risk-loving subjects are also more impatient, a combination that may favor their involvement in risky behaviors. The significant correlation between impatience and attitude toward risk is also reassuring for the goodness of our measure of risk aversion, supporting the absence of alcohol’s effects in increasing risk-taking as a genuine result. Alcohol does not affect the interplay between risk tolerance and impatience, as shown by the fact that the coefficient of the interaction between risk aversion and the intoxication level is fairly close to zero (results are not reported to save space but are available upon request).

Column (5) introduces three interaction terms between *MBAC* and the time dummies (MBAC*1D, MBAC*7D and MBAC*8D), in order to determine whether the pharmacological effect of alcohol on the time discount factor varies across future dates. The results suggest that, relative to the present, subjects with high measured BAC do not discount a one-day payment postponement differently from other subjects, but they discount a seven- or eight-day postponement significantly more than other subjects do.

In order to conduct a similar analysis about the differential effects of alcohol misperception, Column (6) adds three interaction terms between *MPBAC* and the time dummies (MPBAC*1D, MPBAC*7D and MPBAC*8D), but none of them is significant, suggesting that the overestimation of one’s alcohol intoxication does not significantly raise impatience. As a robustness check we replicated the analysis in Table 6 in a cross-section framework using a summary measure for subjective impatience, namely the Area-Under-the-Curve (AUC) of the three choices, as dependent variable. Results (included in S1 Additional Regressions) confirm a positive and highly significant relationship between impatience, measured by AUC, and alcohol intoxication. Treatment dummy regressions confirm the results of both panel and cross-sectional analysis, showing a significant pharmacological effect of alcohol (which makes subjects more impatient), but no significant placebo effect.

### Alcohol and Altruistic Preferences

Phases 3 and 4 of the experiment assess the effect of alcohol on altruism. The descriptive statistics in Table 1 reveal two main observations. First, donations to the humanitarian group MSF are substantially higher than those made to the non-humanitarian group LV in the ALC-P and NO-ALC treatments (*p* = 0.0068 and *p* = 0.0002, respectively, according to Mann-Whitney tests), but not in the ALC-T treatment (*p* = 0.3122). Second, overall donations made by subjects in the NO-ALC group are systematically higher than those made by subjects in the ALC treatments. Such differences reach conventional significance levels according to a battery of Mann-Whitney tests, with the exception of the donations to MSF in the ALC-P and NO-ALC treatments (p = 0.1664). More specifically, the p-values is 0.0025 for donations to MSF comparing ALC-T and NO-ALC. As far as donations to LV are concerned, p-values are 0.0120 (NO-ALC vs. ALC-T) and 0.0232 (NO-ALC vs. ALC-P), respectively.

Table 7 reports random-effect estimates from a panel model that uses the amounts each individual donated to the two NGOs as a longitudinal variable, *Altruism*, with standard errors clustered at the individual level.

**Table 7:** Alcohol and Altruistic Preferences.

Dependent variable	*Altruism*	*Altruism*	*Altruism*	*Altruism*	*Altruism*	*Altruism*
	(1)	(2)	(3)	(4)	(5)	(6)
*MBAC*	-1.8276	-1.8276	-2.0255	-2.0060		
	(2.4772)	(2.4855)	(2.3832)	(2.3543)		
*MPBAC*	-.6253	-.6253	.4831	.4537	.4537	
	(2.1045)	(2.1115)	(2.0939)	(2.0264)	(2.0334)	
*NO-ALC*	2.2787	2.2787	1.6529	1.5002	1.5002	1.5002
	(1.4113)	(1.4160)	(1.3925)	(1.4029)	(1.4077)	(1.4126)
*MSF*		3.8909***	3.8909***	3.8909***	4.7510***	5.0087***
		(.6440)	(.6484)	(.6506)	(.7990)	(.9019)
*Age*			.1669*	.1645*	.1645*	.1645*
			(.0965)	(.0974)	(.0977)	(.0980)
*Female*			2.3391**	2.2824**	2.2824**	2.2824**
			(.9573)	(.9643)	(.9676)	(.9710)
*WTP*				.0483	.0483	.0483
				(.1058)	(.1061)	(.1065)
MBAC*MSF					-4.7069*	-4.7284*
					(2.6349)	(2.6298)
MBAC*LV					.6948	.7163
					(2.3644)	(2.3879)
MPBAC*MSF						-.6126
						(2.3856)
MPBAC*LV						1.5200
						(2.0336)
*Constant*	4.9621***	3.0167***	-1.8183	-1.9131	-2.3431	-2.4720
	(1.2570)	(1.1699)	(2.3748)	(2.3330)	(2.3331)	(2.3515)
Observations	154	154	154	154	154	154
Overall R-Square	.0827	.2141	.2765	.2782	.2968	.2991

*Notes:* Random effect panel estimate. The dependent variable, *Altruism*, is the donation to two NGOs (Mèdecins Sans Frontiéres and LaVoce, see Phases 3 and 4 of the experiment), constructed as a longitudinal variable with two observations for each individual. *MSF* is a dummy for donations to Mèdecins Sans Frontiéres, and MBAC*MSF and MPBAC*MSF are its interactions with *MBAC* and *MPBAC*. The other regressors are defined in the notes to Table 3. Significance level (***: 1%; **: 5%; *: 10%) based on robust standard errors (reported in parenthesis), clustered at the individual level (77 clusters).

Column (1) confirms that donations are higher in the NO-ALC group, but the difference is not statistically significant. The ALC experiment shows a negative association of donations with both *MBAC* and *MPBAC*, but again the coefficients are not significantly different from zero. Column (2) adds a project dummy for MSF, confirming that experimental subjects are significantly and substantially more willing to donate to MSF than to LV. Controlling for age and gender (Column 3) confirms the previous results and shows that donations are significantly higher for older subjects and for females, a result that is common in the literature. Including subjects’ willingness to pay as an additional explanatory variable (Column 4) does not change the results.

To qualify the previous results, the last two columns of Table 7 introduce the interaction terms between measured and misperceived BAC on the one hand, and the *MSF* and *LV* dummies on the other hand. While alcohol does not significantly alter donations to LV, it significantly reduces the amount given to MSF. This reduction in donations to the humanitarian project is attributable to the pharmacological effect of alcohol, rather than the placebo effect, and may signal a lowering attachment to social norms. A Wald test cannot reject the null hypothesis that *MBAC*+*MPBAC* = 0 from Column 1 to 4 (*p* > 0.5 in all specifications), as well as the null hypothesis that MBAC*MSF+MPBAC*MSF = 0 in Column 6 (*p* = 0.1887). This confirms that neither measured nor perceived alcohol intoxication are significant for altruism in general, and that the specific effect of reducing altruism towards humanitarian causes is due to measured and not to perceived alcohol intoxication. Treatment dummy regressions confirm that alcohol intoxication has no significant effect on altruism in general, whether pharmacological or placebo (at least once controlling for age and gender), but it has a specific pharmacological effect of reducing altruism towards humanitarian causes.

## Discussion

In the last two decades, social scientists have devoted substantial effort to studying the behavioral effects of alcohol consumption. In line with the anecdotal evidence, studies generally support the conclusion that alcohol abuse is positively associated with risk-seeking and impatience. This study contributes to the understanding of the effects of alcohol intoxication (measured by BAC) on time preferences, risk-taking and altruism. We do so providing causal evidence gathered by means of a laboratory experiment that substantially reduces self-selection into the treatment and excludes the possibility that results are driven by reverse causality.

Our results confirm the findings of [16] that males’ risk aversion is not affected by alcohol intoxication, even using a different elicitation method and in an experimental setting that is designed to control for self-selection. By contrast, we find some evidence that female’s risk aversion increases with alcohol intoxication. We fail to detect a significant impact of alcohol on the general propensity to donate, although we find a significant reduction in altruism towards humanitarian causes. We also detect a substantial and robust positive relationship between alcohol intoxication and impatience, as measured by the amount that subjects require to postpone the payment to a future date.

Additional observations qualify our experimental findings. First, our results reveal that the impact of alcohol intoxication on impatience is essentially pharmacological and goes beyond the expectation-mediated effects. The effect we identify is stronger than what found in other randomized experiment, while the direction of our results is in line with [39]. We believe that the stronger and more salient incentives used in our design account, at least in part, for the differences between our results and those obtained by other experiments. For instance, [39] use the Experiential Discounting Task (EDT) to elicit time preferences, where choices are delayed by a few seconds only and are diluted by a probabilistic component because later rewards are uncertain. [38], who do not find significant effects of alcohol intoxication, uses a within-subject design in which the task requires subjects to make more than one hundred decisions between different combinations of current and delayed rewards. The pay-one-at-random mechanism used in the mentioned study implies that each decision is made under weak monetary incentives.

Second, we contribute to the literature on the interplay between risk-taking and time preferences. The fact that the future is uncertain suggests that the more one dislikes uncertainty, the more one wants to be compensated not only for facing risk but also for postponing gratification. Therefore, a positive correlation between risk aversion and impatience should be observed. On the other hand, impulsivity can be regarded as a general trait that includes both an inability to delay gratification (impatience) and a tendency to take risks. In this case, we should expect a positive correlation between impatience and risk taking (this is the typical interpretation of impulsivity in the psychology literature). The existing evidence is mixed. For instance, [50] and [38] report that risk aversion increases with impatience, while [51] find opposite results, a negative and significant correlation between subjects’ degrees of risk aversion and their (implicit) discount factors. By using data on risk and time preferences at the individual level and after controlling for the effects of alcohol intoxication, our study reveals the presence of a significant inverse relationship between impatience and risk aversion. Our results lend support to [52] who convincingly argue that how individuals discount delayed and probabilistic outcomes involves different types of impulsivity without being necessarily uncorrelated. In fact, the correlation we find between risk taking and impatience is in line with the results of [53] reported by [52].

Our results also contribute to a growing field of studies in behavioral economics, which, building on evidence from both psychology and the neuro-sciences, describes the decision process as a compromise between deliberation and emotions. According to dual-self models, the relative weight of the deliberative and of the emotional selves in decision-making is affected by an individual’s cognitive load, which reduces the ability to exert willpower and thus shifts the weight toward the impulsive and emotional self (see for instance [54, 55]. [56] argue that while the affective system has initial control, the deliberative system can influence behavior through the exertion of willpower). If one accepts that alcohol reduces the ability to exert willpower and self-control, its effects can be interpreted as analogous to those of cognitive load: alcohol intoxication results in decisions being determined more by emotions and less by deliberation. According to this view, by exploiting an exogenous variation in blood alcohol concentration, our experiment may shed light on the behavioral implications of the emotional self. It provides prima facie evidence that the emotional self is, indeed, impulsive and lead by immediate drives rather than by the calculation of future consequences, thereby inducing individuals to make decisions that they may regret later (partially in line with our results, a recent experimental study by [57] shows that cognitive load increases both small-stakes risk aversion and short-run discounting).

It may be useful to point out three directions for future research, that arise from limitations of the present study. First, replicating the analysis with a larger and more varied sample would increase the precision of the estimates and the external validity of the results. Second, the average alcohol intoxication level in our treatment was around 0.6. We conjecture that, at higher intoxication levels, alcohol may have an even stronger effect on impatience and a significant impact on risk aversion, but more evidence is required to validate such conjecture. Third, in our experiment alcohol intoxication significantly raises impatience through a pharmacological effect, but overall variations in intoxication explain only a small part of the variance of impatience. Moreover, when we disregard the intensive margin (the degree of intoxication) and simply exploit the extensive margin (comparing subjects in the treated and in the placebo group), the significance of the effect of alcohol on impatience drops from 5% to 10%. These aspects of our results are likely to be a direct consequence of the relatively small sample size and the low average intoxication levels in ALC-T. Alternatively, they might be due to the fact that the “true” effect of alcohol on impatience is small. Replication and further experimental studies across different populations and across different intoxication levels are called for to extend and deepen our results, check their external validity and get additional insights.

## Supporting Information

S1 Additional Regressions Additional regressions and robust checks. (TEX)

S1 Experimental Instructions English translation of the experimental instructions. (TEX)
